# Supplementary material for: A large phylogenetic tree for euphyllophytes
Source: Appl Plant Sci. 2026 Nov 10:e70092. Online ahead of print. doi: 10.1002/aps3.70092 (PMC13752582; doi:10.1002/aps3.70092)
Supplement: Supplementary file 1 [file APS3-9999-e70092-s002.docx]

**Appendix S1.** Fossil calibrations used in the backbone tree spanning euphyllophytes.

| **Fossil** | **Node** | **Age (Ma)** | **Reference** |
| --- | --- | --- | --- |
| *Remillia* | Euphyllophyte Crown | 388 (Min) | Dannenhoffer and Bonamo, 1989 |
| *Baragwanathia* | Euphyllophyte Crown | 423 (Max) | Garratt, 1978 |
| Cordiatales | Spermatophyte Crown | 318 (Min) | Phillips, 1980; Taylor et al., 2009 |
| *Elkinsia polymorpha* | Spermatophyte Crown | 366.8 (Max) | Rothwell et al., 1989 |
| Secondary constraint | Angiosperm Crown Node | 154 (Max) | Zuntini et al., 2024 |
| Archaeocalamites | Equisetales stem node | 323.2 (Min) | Bateman, 1991 |
| *Grammatopteris* | Osmundales stem node | 299.0 (Min) | Skog, 2001 |
| *Szea sinensis* | Gleicheniaceae stem node | 270.6 (Min) | Yao and Taylor, 1988 |
| *Stachypteris* | *Lygodium* stem node | 167.7 (Min) | Van Konijnenburg-Van Cittert, 1981; Wikström et al., 2002 |
| Regnellites | Marsileaceae stem node | 140.2 (Min) | Yamada and Kato, 2002 |
| Kuylisporites | *Alsophila* + *Cyathea* stem node | 93.5 (Min) | Collinson, 2001 |
| Glomerisporites | *Azolla* stem node | 83.5 (Min) | Batten et al., 1998 |
| *Acrostichum* | *Acrostichum* stem node | 65.5 (Min) | Bonde and Kumaran, 2002 |
| *Woodwardia gravida* | *Woodwardia* stem node | 55.8 (Min) | Wilf et al., 1998; Collinson, 2001 |
| *Makotopteris princetonensis* | *Athyrioids* stem node | 37.2 (Min) | Stockey et al., 1999 |
| *Protodrynaira sp.* | Polypodiaceae stem node | 33.9 (Min) | Vikulin and Bobrov, 1987 |
| *Polypodium radonii* | Polypodium stem node | 25.6 (Min) | Kvaček, 2001 |
| *Coniopteris lunzenis* | Polypodiales + Cyathaeales stem node | 228 (Min) | Dobruskina, 1994; Schneider and Kenrick, 2001; Schneider et al., 2004 |

**Appendix S2.** Fossil calibrations used in the backbone tree spanning spermatophytes.

| **Fossil** | **Node** | **Age (Ma)** | **Reference** |
| --- | --- | --- | --- |
| Secondary constraint | Spermatophyte crown | Node age estimated in backbone tree spanning euphyllophytes |  |
| *Cordaixylon iowensis* | Acrogymnospermae crown | 306.2 (Min) | Trivett 1992; Peppers, 1996 |
| *Araucarities rudicula* | Araucariaceae + Podocarpaceae crown | 213.0 (Min) | Axsmith and Ash, 2006 |
| *Austrohamia minuta* | Cupressaceae crown | 182.7 (Min) | Escapa and Bodnar, 2017 |
| *Fokienia ravenscragensis* | *Fokienia* + *Platycladus* crown | 61.1 (Min) | McIver and Basinger, 1990; McIver, 1992 |
| *Metasequoia* | Sequoioideae crown | 93.9 (Min) | LePage et al., 2005 |
| *Crossozamia* | Cycadales crown | 270.0 (Min) | Gao and Thomas, 1989a, b |
| *Spermopteris* | Cycadales stem | 298.9 (Min) | Mamay, 1976 |
| *Cratonia cotyledon* | Gnetaceae + Welwitschiaceae crown | 114.0 (Min) | Rydin et al., 2002 |
| *Ephedra drewriensis* | *Ephedra* stem | 125.0 (Min) | Rydin et al., 2006 |
| Polyplicate pollen | Gnetales stem | 265.1 (Min) | Wilson, 1962 |
| *Eathiestrobus mackenziei* | Pinaceae crown | 151.1 (Min) | Rothwell et al., 2012 |
| *Picea burtonii* | *Picea* stem | 132.9 (Min) | Klymiuk and Stokey, 2012 |
| *Palaeotaxus redivia* | Taxaceae stem | 197.0 (Min) | Florin, 1958 |
| Secondary constraint | Angiosperm crown node | 154 (Max) | Zuntini et al., 2024 |
| *Montsechia vidalii* | *Ceratophyllum* stem | 127.2 (Min) | Zuntini et al., 2024 |
| *Changii indicum* | Ehrhartoideae stem | 66.0 (Min) | Zuntini et al., 2024 |
| *Teixeiraea lusitanica* | Ranunculales stem | 110.8 (Min) | Zuntini et al., 2024 |
| *Hyrcantha decussata* | Eudicots stem | 125 (Min) | Zuntini et al., 2024 |
| *Dressiantha bicarpellata* | Brassicales stem | 86.3 (Min) | Zuntini et al., 2024 |

**REFERENCES**

Axsmith, B. J., and S. R. Ash. 2006. Two rare fossil cones from the Upper Triassic Chinle Formation in Petrified Forest National Park, Arizona, and New Mexico. *Museum of Northern Arizona Bulletin* 62: 82–94.

Bateman, R. M. 1991. Palaeobiological and phylogenetic implications of anatomically-preserved archaeocalamites from the Dinantian of Oxroad Bay and Loch Humphrey Burn, southern Scotland. *Palaeontographica Abteilung B Band* 223: 1–59.

Batten, D. J., M. E. Collison, and A. P. R. Brain. 1998. Ultrastructural interpretation of the Late Cretaceous megaspore *Glomerisporites pupus* and its associated microspores. *American Journal of Botany* 85: 724–735.

Bonde, S. D., and K. P. N. Kumaran. 2002. The oldest macrofossil record of the mangrove fern *Acrostichum* L. from the Late Cretaceous Deccan Intertrappean beds of India. *Cretaceous Research* 23: 149–152.

Collinson, M. E. 2001. Cainzoic ferns and their distribution. *Brittonia* 53: 173–235.

Dannenhoffer, J. M., and P. M. Bonamo. 1989. *Rellimia thomsonii* from the Givetian of New York: Secondary growth in three orders of branching. *American Journal of Botany* 76: 1312–1325.

Dobruskina, I. A. 1994. Triassic floras of Eurasia. Verlag der Österreichischen Akademie der Wissenschaften, Vienna, Austria.

Escapa, I., and J. Bodnar. 2017. Towards a whole plant reconstruction for Austrohamia (Cupressaceae): New fossil wood from the Lower Jurassic of Argentina. *Review of Palaeobotany and Palynology* 234: 186–197.

Florin, R. 1958. On Jurassic taxads and conifers from north-western Europe and Eastern Greenland. *Acta Horti Bergiani* 17: 257–402.

Gao, Z., and B. A. Thomas. 1989a. Occurrence of earliest cycads in the Permian of China and its bearing on their evolution. *Chinese Science Bulletin* 34: 766–769.

Gao, Z., and B. A. Thomas. 1989b. A review of fossil cycad megasporophylls, with new evidence of Crossozamia Pomel and its associated leaves from the Lower Permian of Taiyun, China. *Review of Palaeobotany and Palynology* 60: 205–223.

Garratt, M. J. 1978. New evidence for a Silurian (Ludlow) age for the earliest Baragwanathia flora. *Alcheringa* 2: 217–224.

Klymiuk, A. A., and R. A. Stockey. 2012. A lower Cretaceous (Valanginian) seed cone provides the earliest fossil record for Picea (Pinaceae). *American Journal of Botany* 99: 1069–1082.

Kvaček, Z. 2001. A new fossil species of *Polypodium* (Polypodiaceae) from the Oligocene of northern Bohemia (Czech Republic). *Feddes Repert* 112: 159–177.

LePage, B. A., H. Yang, and M. Matsumoto. 2005. The evolution and biogeographic history of *Metasequoia*. In B. A. LePage, C. J. Williams, and H. Yang [eds.], *The Geobiology and Ecology of Metasequoia*, 3–114. Springer, Dordrecht, the Netherlands.

Mamay, S. H. 1976. Paleozoic origin of cycads. *Geological Survey Professional Paper* 934. U.S. Government Printing Office, Washington, D.C., USA.

McIver, E. E. 1992. Fossil *Fokienia* (Cupressaceae) from the Paleocene of Alberta, Canada. *Canadian Journal of Botany* 70: 742–749.

McIver, E. E., and J. E. Basinger. 1990. Fossil seed cones of *Fokienia* (Cupressaceae) from the Paleocene Ravenscrag Formation of Saskatchewan, Canada. *Canadian Journal of Botany* 68: 1609–1618.

Peppers, R. A. 1996. Palynological correlation of major Pennsylvanian (Middle and Upper Carboniferous) chronostratigraphic boundaries in the Illinois and other coal basins. *Geological Society of America* 188: 1–111.

Phillips, T. L. 1980. Stratigraphic and geographic occurrences of permineralized coal-swamp plants - Upper Carboniferous of North America and Europe. *In* D. L. Dilcher and T. N. Taylor [eds.], Biostratigraphy of fossil plants, 93–118. Hutchinson and Ross, Stroudsburg, Pennsylvania, USA.

Rothwell, G. W., S. E. Scheckler, and W. H. Gillespie. 1989. *Elkinsia* gen. nov., a late Devonian gymnosperm with cupulate ovules. *Botanical Gazette* 150: 170–189.

Rothwell, G. W., G. Mapes, R. A. Stockey, and J. Hilton. 2012. The seed cone *Eathiestrobus* gen. nov.: Fossil evidence for a Jurassic origin of Pinaceae. *American Journal of Botany* 99: 708–720.

Rydin, C., B. Mohr, and E. M. Friis. 2002. *Cratonia cotyledon* gen. et sp. nov.: A unique Cretaceous seedling related to *Welwitschia*. *Proceedings of the Royal Society B* 270: S29–S32.

Rydin, C., K. R. Pedersen, P. R. Crane, and E. M. Friis. 2006. Former diversity of *Ephedra* (Gnetales): Evidence from Early Cretaceous seeds from Portugal and North America. *Annals of Botany* 98: 123–140.

Schneider, H., and P. Kenrick. 2001. An Early Cretaceous rot-climbing epiphyte (Lindsaeaceae) and its significance for calibrating the diversification of polypodiaceous ferns. *Review of Palaeobotany and Palynology* 115: 33–41.

Schneider, H., E. Schuettpelz, K. M. Pryer, R. Cranfill, S. Magallón, and R. Lupia. 2004. Ferns diversified in the shadow of angiosperms. *Nature* 428: 553–557.

Skog, J. E. 2001. Biogeography of Mesozoic leptosporangiate ferns related to extant ferns. *Brittonia* 53: 236–269.

Stockey, R. A., H. Nishida, and G. W. Rothwell. 1999. Permineralized ferns from the Middle Eocene Princeton Chert. I. *Makopteris princetonensis* Gen. et Sp. Nov. (Athyriaceae). *International Journal of Plant Sciences* 160: 1047–1055.

Taylor, T. N., E. L. Taylor, and M. Krings. 2009. Paleobotany: The biology and evolution of fossil plants. Academic Press, Burlington, Massachusetts, USA.

Trivett, M. L. 1992. Growth, architecture, structure, and relationships of *Cordaixylon iowensis* nov comb (Cordaitales). *International Journal of Plant Sciences* 153: 273–287.

Van Konijnenburg-Van Cittert, J. H. A. 1981. Schizaeceous spores *in situ* from the Jurassic of Yorkshire, England. *Review of Palaeobotany and Palynology* 33: 169–181.

Vikulin, S. V., and A. E. Bobrov. 1987. New fossil genus *Protodrynaria* (Polypodiaceae) from the Paleogene Flora of Tim (the South of the Middle Russian Upland). *Botanicheskii zhurnal* 72: 95–98.

Wikström, N., P. Kenrick, and J. C. Vogel. 2002. Shizaceae: A phylogenetic approach. *Review of Palaeobotany and Palynology* 119: 35–50.

Wilf, P., K. C. Beard, K. S. Davies-Vollum, and J. W. Norejko. 1998. Portrait of a Late Paleocene (Early Clarkforkian) terrestrial ecosystem: Big Multi Quarry and associated strata, Washakie Basin, Southwestern Wyoming. *Palaios* 13: 514–532.

Wilson, L. R. 1962. Permian plant microfossils from the Flowerpot Formation, Greer County, Oklahoma. *Oklahoma Geological Survey Bulletin* 49: 5–50.

Yamada, T., and M. Kato. 2002. *Regnellites nagashimae* gen. et sp. nov., the Oldest Macrofossil of Marsileaceae, from the Upper Jurassic to Lower Cretaceous of Western Japan. *International Journal of Plant Sciences* 163: 715–723.

Yao, Z., and T. N. Taylor. 1988. On a new gleicheniaceous fern from the Permian of South China. *Palaeobotany and Palynology* 54: 121–134.

Zuntini, A. R., T. Carruthers, O. Maurin, P. C. Bailey, K. Leempoel, G. E. Brewer, N. Epitawalage, et al*.* 2024. Phylogenomics and the rise of the angiosperms. *Nature* 629: 843–850.
